# Lectins as Promising Therapeutics for the Prevention and Treatment of HIV and Other Potential Coinfections

**DOI:** 10.1155/2018/3750646

**Published:** 2018-05-08

**Authors:** Milena Mazalovska, J. Calvin Kouokam

**Affiliations:** ^1^Department of Pharmacology and Toxicology, University of Louisville School of Medicine, Louisville, KY 40202, USA; ^2^Center for Predictive Medicine, University of Louisville, Louisville, KY 40202, USA; ^3^James Graham Brown Cancer Center, University of Louisville School of Medicine, Louisville, KY 40202, USA

## Abstract

Human immunodeficiency virus-acquired immunodeficiency syndrome (HIV/AIDS) remains a global health problem. Current therapeutics specifically target the viral pathogen at various stages of its life cycle, although complex interactions between HIV and other pathogenic organisms are evident. Targeting HIV and concomitant infectious pathogens simultaneously, both by therapeutic regimens and in prevention strategies, would help contain the AIDS pandemic. Lectins, a ubiquitous group of proteins that specifically bind glycosylated molecules, are interesting compounds that could be used for this purpose, with demonstrated anti-HIV properties. In addition, potential coinfecting pathogens, including other enveloped viruses, bacteria, yeasts and fungi, and protozoa, display sugar-coated macromolecules on their surfaces, making them potential targets of lectins. This review summarizes the currently available findings suggesting that lectins should be further developed to simultaneously fight the AIDS pandemic and concomitant infections in HIV infected individuals.

## 1. Introduction

Human immunodeficiency virus (HIV) infection remains a leading public health problem, due to the rapid mutation of the virus, resistance to existing therapies, lack of a vaccine, and inadequate use of physical prophylaxis, for example, resistance to condom use for cultural or religious reasons [1]. Despite recent advances in acquired immunodeficiency syndrome (AIDS) therapy and the available prevention methods, HIV infection continues to spread worldwide with about 5,500 new infections every day [2]. In many developing countries, notably in sub-Saharan Africa, AIDS is considered a disaster because of its devastating socioeconomic effects [3].

There have been tremendous advances over the past years in the standard use of antiretroviral drugs (ARVs) for the treatment and prevention of HIV infection [4]. Indeed, current ARVs greatly improve the clinical outcomes of HIV positive individuals and limit disease transmission with improved safety, elevated tolerability, and high potency, especially if used early in the infection [5].

One of the most recent tools for HIV prevention is preexposure prophylaxis (PrEP), whereby individuals are prescribed daily doses of antiviral drugs in anticipation of exposure, thus lowering the risk of infection. Truvada was the first FDA approved drug for HIV PrEP in an attempt to reduce and prevent HIV infection among high risk individuals; its consistent intake is highly effective in preventing HIV infection [6]. Despite the increasing awareness of health benefits associated with PrEP, its use remains low especially among women [7]. Like other preventive measures, the efficacy of PrEP is highly related to adherence, heavily relying on individuals taking pills daily for long periods of time. Even though the use of antiviral drugs has increased the lifespan of individuals with HIV, they are still more susceptible to other opportunistic infections that are potentially lethal [8]. It was shown that individuals using PrEP are more prone to other sexually transmitted diseases, mainly from unprotected sex and increased number of sexual partners [9]. In addition, opportunistic infections also accelerate AIDS progression. All things considered, the high cost of antiviral drugs and the risk of opportunistic infections in individuals with HIV demand the development of new antiviral drugs that could simultaneously target other pathogens.

Lectins are a unique and heterologous class of proteins with the ability to recognize and reversibly bind a variety of sugar structures present on the cell surface [10]. They are found in a wide range of organisms, from viruses and bacteria to animals, plants, and humans [11]. The broad distribution in nature shows that lectins have important biological functions in the corresponding organisms, including cell-cell interactions, protection from pathogens, cell adhesion, and intracellular translocation of glycoproteins, also acting as storage proteins [12–14]. Interestingly, multiple lectins have been shown to neutralize a number of viruses, including HIV, making them attractive targets for the development of novel antiviral drugs [15]. Moreover, considering their mechanisms of action, increasing evidence suggests that lectins could also target other groups of pathogens, including prokaryotic and eukaryotic organisms. This review summarizes the currently available findings demonstrating that lectins can be considered promising means to combat the HIV pandemic as well as concomitant infections in HIV infected individuals, providing affordable regimens for prevention and/or treatment.

## 2. Lectins Can Be Used for the Treatment and/or Prevention of HIV Infection

Even though antiretroviral therapy has successfully improved health outcomes in HIV infected individuals, especially those living in industrialized countries, a high percentage of patients still do not have access to ARVs in poor areas (e.g., sub-Saharan Africa), where the disease burden is the highest [16]. Although availability is no longer a crucial issue, lack of trained clinicians and increased prevalence of associated diseases, such as tuberculosis, have impacted the management of HIV in sub-Saharan Africa [16]. Moreover, the fact that ARVs are associated with risk of developing other infectious diseases demands the development of new antiviral drugs to include in a comprehensive package designed to contain the AIDS pandemic.

Entry inhibitors would constitute important additions to the currently available weapons against HIV-1 infection [17, 18]. Indeed, recent approaches have led to the design of active compounds interfering with entry events. For example, T-20, T-2410, and T-2429 inhibit gp41 mediated virus entry [19–21]; meanwhile, Maraviroc belongs to a separate class of antiretroviral agents that target a host protein, the chemokine coreceptor CCR5, rather than a viral molecule [22].

HIV-1 entry into target cells is mediated by binding of its envelope glycoprotein to cell surface receptors. The envelope glycoprotein gp160 is originally synthesized as a single, glycosylated, polyprotein precursor, which is then cleaved by a cellular protease to yield two subunits, including gp120 (responsible for viral binding to CD4 receptor and a coreceptor, namely, CCR5 or CXR4) and gp41, which mediate viral fusion to the cell [23]. HIV uses gp120 to bind C-type lectin receptor (CLR) on macrophages as well as DC-SIGN, a C-type mannose-specific lectin, expressed by dendritic cells [24].

Due to the glycosylated nature of viral envelope proteins, carbohydrate-binding proteins have been considered potential anti-HIV agents that would block the host-virus interaction at its earliest stage, eventually preventing the establishment of the provirus [25]. Lectins with antiretroviral activities have been identified and isolated from animals [26], plants [27], halobios, and microorganisms [28] and could serve as anti-HIV natural products.

### 2.1. Mechanisms of Anti-HIV Activity of Lectins

Many enveloped viruses are covered by virally encoded glycoproteins displayed on the surface. In the case of HIV-1, the envelope glycoproteins gp120 and gp41 are heavily glycosylated, and N-linked carbohydrates might make up to 50% of the total molecular weight of gp120 [29]. All the potential 24 N-linked glycosylation sites are utilized in gp120, 13 of which contain complex-type oligosaccharides while 11 primarily comprise high-mannose-type and/or hybrid-type oligosaccharide structures [30, 31]. The associated oligosaccharides contain mannose, galactose, N-acetyl-glucosamine (GlcNAc), N-acetyl-galactosamine (GalNAc), L-fucose, and sialic acid in their branches [31]. The most credible mechanism proposed for virus-cell attachment involves the interaction between positively charged regions of the viral-envelope glycoproteins and negatively charged heparan sulphate proteoglycans. The fusion process starts with binding of gp120 to the cell-surface CD4 antigen. These entry events are vulnerable to agents that specifically and strongly interact with the glycans since they may disturb the association of viral envelope proteins with host cell receptors, that is, gp120 and CD4, respectively [18, 32]. It is believed that these agents alter the efficient interaction between gp120 (or gp41) and its (co)receptors through steric hindrance, prevention of necessary conformational changes of* env,* and/or cross-linking of several glycans present on* env* and/or the target cell [33]. Lectins, which are carbohydrate-binding proteins, possess such binding properties. Indeed, sound anti-HIV effects are attributed to lectins with specific recognition for mannose (Man) and/or GlcNAc [8, 33]. A number of plant and microbial lectins have been researched in recent years, including griffithsin (GRFT), actinohivin (AH), concanavalin-A (ConA), cyanovirin-N (CV-N), microvirin (MVN), and banana lectin (BanLec). Generally speaking, these lectins contain multiple sugar-binding sites allowing them to form multivalent interactions with gp120. Such interactions confer to lectins the ability to neutralize a broad range of lab-adapted and clinically isolated strains of HIV-1 and HIV-2. The following are a few examples of lectins that have been investigated for their antiretroviral activities.

### 2.2. Cyanovirin-N (CV-N)

Cyanovirin-N (CV-N) is a lectin that was firstly isolated from the cyanobacterium* Nostoc ellipsosporum,* in an attempt to discover anti-HIV agents from natural extracts [19]. The CV-N lectin consists of 101 amino acids with a molecular weight of 11 kDa and contains two carbohydrate-binding sites with specificity towards the terminal *α*1,2-mannose sugars on high-mannose glycans [34]. CV-N has broad neutralization activity in the low nanomolar range against primary isolates and laboratory-adapted strains of HIV-1 and HIV-2, simian immunodeficiency virus (SIV), and feline immunodeficiency virus [35]. This broad activity of CV-N is due to high affinity of binding to gp120, essential for its anti-HIV effects. The mechanism of action of CV-N is still not quite understood, but it is believed that the antiviral activity against HIV occurs after virus-cell attachment or at a post-CD4-binding step in the entry process [36]. Unfortunately, CV-N treatment results in cell activation, mitogenicity, and increased cytokine production in human PMBC [37].

### 2.3. Microvirin (MVN)

Microvirin (MVN) is a lectin with 108 amino acids (14 kDa) isolated from the cyanobacterium* Microcystis aeruginosa*. MVN is a monomer in solution with one sugar binding site that also recognizes terminal *α*1,2-mannose sugars [38]. Like CV-N, MVN has the ability to neutralize a wide range of laboratory-adapted strains and clinical isolates with low nanomolar potency in most HIV-1 group M clades. Even though MNV shares 33% identity with CV-N, the former lectin is more potent and 50-fold less toxic than CV-N. Therefore, MNV is a very attractive lectin as a topical microbicide because of its ability to inhibit HIV-1 and its reduced toxicity profile [39].

### 2.4. Actinohivin (AH)

Actinohivin (AH) is an anti-HIV lectin isolated from the culture broth of the actinomycete* Longispora albida * [40]. The protein is a single polypeptide with a molecular weight of 12.5 kDa and 114 amino acids that form three sugar-binding pockets with LD-QXW motifs [28]. These three segments are highly conserved and necessary for both B- and T-tropic antisyncytium formation activity [41]. Tanaka et al. reported that AH specifically binds to high-mannose-type glycans [37]. Additionally, AH has been shown to have affinity only to glycoproteins with multiple high-mannose glycans, unlike CV-N which can bind a single high-mannose glycan attached to a protein. Also unlike CV-N, AH does not show cytopathic or mitogenic effects. Based on previous studies AH has the potential to be developed as a microbicide drug since it can effectively inhibit HIV-1 and HIV-2 with low IC50 values (2–110 nM), block syncytium formation, and display neutralization activity against laboratory-adapted strains with minimal variation in antiviral activity [42].

### 2.5. BanLec

BanLec is a member of the family of the jacalin-related lectins. The lectin isolated from the fruit of bananas,* Musa acuminate*, also has affinity towards high-mannose structures. The native lectin is a dimer composed of two identical subunits of 15 kDa containing 141 amino acids [27] and two sugar binding sites each [43]. It was shown that BanLec can inhibit various HIV-1 isolates with different tropisms* in vitro*, with IC50 values in the low nanomolar range. Similar to the above carbohydrate-binding proteins, BanLec inhibits HIV infection at the viral entry step by binding to high-mannose structures present on the heavily glycosylated gp120 in a concentration dependent manner, thus preventing attachment of the virus to the cell. Swanson et al. also reported that BanLec is a potent mitogen for murine T-cells, although the effects depend on the mouse strain used. Interestingly, a mutation within the sugar-binding site of BanLec notably reduces its mitogenic activity without affecting HIV neutralization. The new engineered lectin has the potential to be used as a microbicide drug [44].

### 2.6. Griffithsin (GRFT)

Griffithsin (GRFT) is another high-mannose-binding lectin isolated from the marine red algae* Griffithsia *sp. and considered the most potent HIV entry inhibitor to date [45]. The protein contains 121 amino acids and folds into a stable domain-swapped homodimer, with each subunit capable of binding three monosaccharides. GRFT is currently the leading lectin candidate for clinical use as an HIV prophylactic, neutralizing HIV with IC50 in the picomolar range [45]. Indeed, this lectin is more potent than broadly neutralizing antibodies (bNAbs), including 2G12 which also binds to high-mannose-type glycans. GRFT has potent activity against both T-tropic and M-tropic viruses and inhibits clade A, B, and C viruses. Furthermore, it is capable of preventing HIV in human cervical explant tissues with no proinflammatory cytokine production. GRFT has an excellent safety profile when tested in a rabbit vaginal irritancy model [46], with minimal toxicity when administered in single or chronic subcutaneous doses in mice and guinea pigs [47].

## 3. Lectins Are Active against Viruses Other Than HIV

Considering its clinical importance, the majority of studies assessing antiviral lectins have focused on HIV. However, based on their mechanisms of action carbohydrate-binding agents may target a multitude of enveloped viruses which share the feature of heavily glycosylated proteins on their surfaces. Not surprisingly, anti-HIV lectins have been extensively evaluated for their effects on other enveloped viruses (Table 1). For instance,* Aspidistra elatior* lectin (AEL) displays antiviral effects against the enveloped respiratory syncytial virus but also against Coxsackie virus B4, a non-enveloped virus [48], suggesting that lectins may target other viral components than surface glycoconjugates. This notion was confirmed by the protective effects of GRFT in mice infected with vaginal HPV infection, although more pronounced effects were achieved by the carrageenan-GRFT combination; such anti-HPV activity likely occurs via *α*6 integrin internalization as shown* in vitro *[49]. In addition, intraperitoneal GRFT was shown to prevent Japanese encephalitis virus (JEV) entry, both* in vitro* and* in vivo* [50]. Mechanistically, GRFT was recently reported to bind to the glycosylated proteins E and prM of JEV [51]. GRFT was assessed in parallel with other potent anti-HIV lectins from algae, namely, CV-N and scytovirin (SVN), and differential inhibitory activities were found against other enveloped viruses such as gammaretroviruses and deltaretroviruses [52]. A few envelope viruses sensitive to lectins are discussed below.

### 3.1. Lectins Targeting Hepatitis C Virus (HCV)

Presently, 20% of HIV patients are coinfected with HCV [68]. Infection with HCV causes either acute or chronic infection, which can progress to cirrhosis with the need for liver transplantation [69]. Usually, HCV-HIV coinfection is associated with a faster progression to cirrhosis and liver failure and shows poor response to treatment [70, 71]. As the most potent anti-HIV lectin described so far, GRFT has been widely assessed for its activities against other enveloped viruses. GRFT has been shown to mitigate HCV infection of mice harboring human primary hepatocytes in the liver and prevents* in vitro* HCV infection of Huh-7 hepatoma cells [54]. Notably, GRFT binds to the HCV envelope glycoproteins E1 and E2, blocking viral entry into human hepatocytes [72]. In addition to GRFT, other lectins, including those with anti-HIV activities, have the ability to bind and neutralize HCV. HCV is an enveloped virus, like HIV, with two heavily glycosylated glycoproteins that include E1 and E2 [73]. E1 contains 4-5 N-linked glycosylation sites and E2 up to 11, many of which are high-mannose-type glycans that ensure proper folding, attachment, and entry, protecting the virus from neutralizing antibodies [74]. Targeting viral entry with lectins could potentially be a new strategy to combat HCV. Indeed, cyanovirin-N can inhibit HCV infection by binding the N-linked glycans on its surface, preventing the interaction between E2 and the entry receptor CD81 [53]. Other studies demonstrated that CV-N,* Microcystis viridis* lectin (MVL), and* Galanthus nivalis* agglutinin (GNA) inhibit HCV* in vitro*, with IC50 values of 0.6 nM, 30.4 nM, and 11.1 nM, respectively, likely through distinct and complex modes of action [55, 75].

### 3.2. Lectins with Anti-HSV Activities

Another opportunistic viral pathogen in HIV infected individuals is herpes simplex virus type 2 (HSV-2). HSV-2 is sexually transmitted and a leading cause of genital herpes. Some individuals with the virus never show symptoms, while most experience regular outbreaks of painful sores on the genitalia [76]. It is estimated that more than 400 million people are infected with HSV-2, with a high prevalence in Africa [77]. HSV-2 is a major risk factor for acquiring HIV in both men and women. Indeed, having HSV increases the chances of HIV infection 3-fold [78], and the chances are higher in individuals with newly acquired HSV [79]. Multiple studies have reported a biological synergy between these two viruses; a number of them showed that coinfection with HSV induces HIV replication and increases HIV-1 infectiousness [80]. Coinfection with HSV has also been shown to increase genital shedding from both viruses potentially explaining the higher rate of HIV transmissibility in these individuals [81, 82]. It was reported that GRFT confers protection to mice infected with genital HSV-2 likely by preventing cell-to-cell spread, with no significant adverse effects [61]. In addition to GRFT, other lectins have been assessed for their antiherpes activities. For example, CV-N inhibits entry and cell-to-cell spread of HSV-1 with IC50s in the nanomolar range [62]. Furthermore, a mannose-binding lectin from* Typhonium divaricatum* (L.) Decne (family Araceae) displays antiviral effects against HSV-II, as well as Jackfruit lectin (JFL) from* Artocarpus heterophyllus *[63, 64]. Meanwhile,* Polygonatum odoratum* lectin (POL), a GNA-related mannose-binding lectin, also exerts remarkable anti-HSV-II effects [65].

### 3.3. Lectins Targeting Influenza Viruses

Influenza causes high morbidity in HIV positive individuals [83]. Several reports have demonstrated the anti-inhibitory activities of lectins against influenza viruses. For example, a lectin from the red alga* Kappaphycus alvarezii* (KAA-2) inhibits infection by multiple influenza strains with EC50 values in the low nanomolar range, by interfering with virus entry into host cells upon direct binding of hemagglutinin (HA) on the viral envelope [59]. Similarly, a lectin derived from the green alga* Boodlea coacta* (BCA) exerts potent anti-influenza effects by directly binding HA of multiple strains, including the pandemic H1N1-2009 virus [59]. In addition, three other lectins, including ConA,* Lens culinaris* agglutinin (LCA), and peanut agglutinin (PNA), were shown to suppress cell fusion and hemadsorption associated with human parainfluenza virus type 2 (hPIV-2)* in vitro*, mainly by preventing virus adsorption to the cells [84]. In a screen of 12 lectins, the red alga* Eucheuma serra* derived high-mannose binding anti-HIV lectin ESA-2 was very effective in inhibiting influenza virus infection with an EC50 of 12.4 nM [57]. Finally, the mannose-specific* Narcissus tazetta* lectin (NTL) strongly inhibits influenza A (H1N1, H3N2, and H5N1) and influenza B viruses with IC50 values between 0.20 *μ*g/ml and 1.33 *μ*g/ml and markedly reduces plaque formation by the human respiratory syncytial virus (RSV) with an IC50 of 2.30 *μ*g/ml [60].

### 3.4. Lectins with Inhibitory Activities against Coronaviruses

Coronaviruses constitute an important class of human and animal pathogens, and some have been assessed for their susceptibility to lectins. Interestingly, GRFT also prevents SARS coronavirus (SARS-CoV) infection* in vitro* and* in vivo*, through specific binding to its spike glycoprotein, and shows activity against multiple other coronaviruses pathogenic to humans, other mammals, and birds; in mice, these inhibitory effects were accompanied with a specific inhibition of deleterious host immune reactions in response to SARS [85]. The Middle East respiratory syndrome coronavirus (MERS-CoV), another highly pathogenic human coronavirus, is inhibited at the entry level by GRFT to prevent infection* in vitro* [86]. In addition,* Hippeastrum hybrid* agglutinin (HHA), GNA,* Cymbidium* agglutinin (CA), and* Urtica dioica* agglutinin (UDA) demonstrate antiviral activities against coronaviruses* in vitro* and/or* in vivo *[67]. In an impressive screening of 33 plant lectins, remarkable antiviral effects on both SARS-CoV and feline infectious peritonitis virus (FIPV) with EC50 values at low *μ*g/ml levels were observed, with strongest activities found predominantly among mannose-binding lectins [66]. Based on these data, lectins should be included in antiviral strategies to fight SARS coronavirus infections [87].

## 4. Lectins as Potential Candidates for Fighting Pathogenic Organisms Other Than Viruses in HIV Patients

**Figure 1:**
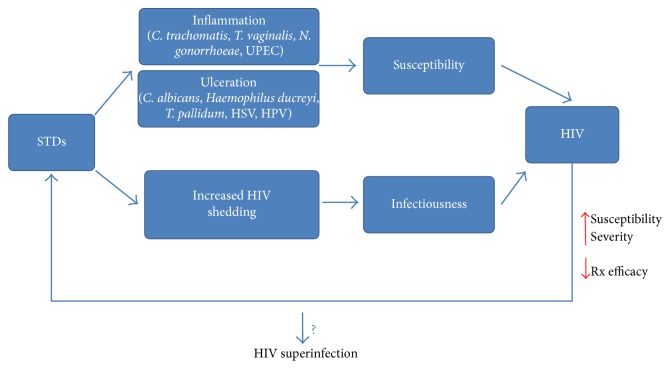
*Bidirectional biological synergy between HIV and sexually transmitted pathogens*. There are complex interactions between HIV infection and sexually transmitted diseases (STDs). Both inflammatory and ulcerative STDs increase HIV susceptibility, while enhancing virus shedding and therefore increasing patient infectiousness. This results in increased HIV burden. Meanwhile, HIV infection increases susceptibility to and the severity of STDs, decreasing also treatment efficacy. This vicious cycle might promote HIV superinfection, which is the reinfection of an individual who already has an established infection with a heterologous HIV strain.* C. trachomatis*:* Chlamydia trachomatis*;* T. vaginalis*:* Trichomonas vaginalis*;* N. gonorrhoeae*:* Neisseria gonorrhoeae*;* UPEC*:* uropathogenic Escherichia coli*;* C. albicans: Candida albicans*;* T. pallidum*:* Treponema pallidum*; HSV: herpes simplex virus; HPV: human papillomavirus; Rx: treatment.

Lectins engage in nonenzymatic and noncovalent, but highly specific, interactions with carbohydrates, binding sugar moieties and oligosaccharides, for example, oligomannose N-linked glycans (NLG) displayed on the viral envelope glycoproteins. Such interactions have important biological effects, and lectins have been widely assessed for their potential activities against enveloped viruses as described above. However, among human pathogens, surface exposed oligosaccharides are not limited to viral envelopes. Indeed, it is widely admitted that glycosylation represents the most important cotranslational and posttranslational modification of proteins in virtually all living organisms [11, 88]. Thus, lectins could theoretically interfere with sugar moieties of glycans displayed on many cell types, including bacteria, yeasts, and parasites. It is known that the incidence of HIV/AIDS is exacerbated in individuals infected with other sexually transmitted pathogens. Both ulcerative (candidiasis, chancroid, syphilis, genital herpes, and genital warts) and inflammatory (chlamydia, gonorrhea, and trichomoniasis) sexually transmitted diseases (STDs) have been shown to increase susceptibility to HIV infection [89–91]. In addition, STDs increase virus shedding in HIV infected patients, rendering them even more infectious [91]. Conversely, in an immune system heavily altered by AIDS, the etiologic agents of STDs have an easier path to cause illness. Therefore, it is reasonable to assume that the biological and epidemiological synergies between HIV and STDs can also promote HIV superinfection, that is, reinfection of an individual who already has an established infection with a heterologous HIV strain (Figure 1). Although the amplitude of HIV superinfection is not entirely known, this complication certainly affects the magnitude of global HIV diversity, which is driven in large part by recombination between viruses [92]. Apart from these sexually transmissible pathogens, other microbes might also take advantage of the weakened immune system of HIV infected patients and cause various diseases and/or further alter the immune system.

Considering the above-mentioned synergy between HIV infection and STDs, in addition to the possibility of superinfection in seropositive individuals, there is an urgent need for novel anti-HIV agents, which would simultaneously target other pathogens. In the case of sexually transmitted organisms, a socioepidemiological rationale was recently proposed for the development of multipurpose prevention technologies [93], since current HIV prevention methods fall well short of needs. This would help tackle both AIDS and other STDs and likely improve the immune system of the affected patients.

### 4.1. Bacterial Organisms Potentially Susceptible to Lectins

Urinary tract infections (UTIs) are among the most common bacterial infections and show elevated prevalence rates in women and HIV positive individuals [94]. Glycoproteins have been shown to be involved in the transmission of bacterial pathogens. For instance, uropathogenic* E. coli* (UPEC), which causes the majority of urinary tract infections (UTIs), need to overcome the constant shear stress of urine flow. This is accomplished by bacterial attachment to the renal epithelium via the attachment organelles types 1 and P fimbriae [95]. More than 90% of all UPEC strains express the adhesin FimH, one of the most described mannose-specific bacterial lectins expressed on the tip of type 1 fimbriae [95–97]. Meanwhile, use of lectins to achieve attachment to host cells is widely spread in bacterial organisms. Indeed, several bacteria bind to the cell glycocalyx for colonization, as is the case for cells in contact with the environment, for example, epithelial cells [98]. Lectins safely delivered to these surfaces targeted by microbial lectins would compete for the binding of bacteria and prevent adhesion, consequently suppressing colonization and infection. Interestingly, we recently demonstrated that GRFT administered parenterally was mainly eliminated through urine [99], indicating that this molecule could help prevent colonization by competition with bacteria for the cell glycocalyx, especially those with mannose type lectins at the surface such as UPEC. Another lectin, Eutirucallin, isolated from the latex of* Euphorbia tirucalli*, also displays antimicrobial activity towards* E. coli* [100].

*Chlamydia trachomatis*, a critical etiologic agent of ocular and genital infections in humans, likely uses carbohydrates for attachment to host cells or entry. Three* C. trachomatis* glycoproteins, including the major outer membrane protein (MOMP; 40-kDa), a 32-kDa outer surface glycoprotein, and an 18-kDa molecule, have been reported [101, 102]. These surface exposed glycoproteins, especially, the MOMP, are critical for attachment and infectivity of* C. trachomatis* to HeLa cells, via their oligomannose-oligosaccharides [101]. It was reported that two plant lectins, including wheat germ agglutinin and* Galanthus nivalis* lectin, can block chlamydial attachment sites and inhibit infection* in vitro* [103, 104].

In* Neisseria meningitides*, surface glycosylated molecules, such as capsule polysaccharide, lipooligosaccharide, and O-linked glycoproteins, have been described, while* N. gonorrhoeae* produces both lipooligosaccharide and O-linked glycoproteins [105]. These glycans are very critical for the host pathogen interactions during infection by* Neisseria* spp. The wheat germ agglutinin, ricin, soybean, and peanut agglutinin have the ability to agglutinate different strains of* Neisseria gonorrhoeae *[106, 107].

Tuberculosis (TB) caused by the bacterium* Mycobacterium tuberculosis* is one of the top 10 causes of mortality worldwide according to the WHO [108]. In addition to killing over one million people yearly, TB is also the leading cause of death among HIV positive individuals [108]. Although TB is treatable, the prevalence of a multiresistant form of TB (MDR-TB) that does not respond to first-line anti-TB drugs calls for the development of new ways to cope with this infection. In* M. tuberculosis*, envelope mannosylated lipoarabinomannans (ManLAMs) and Mtb surface glycoproteins (glycosylated 45-kDa [Apa: alanine- and proline-rich antigenic] and 19-kDa proteins) are considered important antigenic molecules with essential roles in host protection against this pathogen; these antigens may be recognized by dendritic cell-specific intercellular adhesion molecule-3 grabbing nonintegrin (DC-SIGN) and other mannose-specific C-type lectins (C-TLs) [109]. Since* M. tuberculosis* organisms have large amounts of mannosylated cell-surface molecules, an attempt was made to assess whether lectins with potent anti-HIV activity can also inhibit these bacteria. It was reported that CV-N competes with C-type lectins DC-SIGN and mannose receptor for binding to ManLAMs and inhibits the binding of bacteria to dendritic cells. However,* in vivo* findings showed that this binding did not inhibit or delay infection. The authors speculated that such observation could be due to the fact that murine C-type lectins differ from human versions, which requires further investigation [110].

Recently, the novel lectin CasuL produced by* Calliandra surinamensis* was shown to display bacteriostatic effects and reduce biofilm formation by nonresistant and oxacillin-resistant* Staphylococcus* spp. [111]. The plant lectins ConBr and CFL produced by* Canavalia brasiliensis* and* Cratylia argentea*, respectively, induce antimicrobial and immunomodulatory effects in mouse peritoneal macrophages upon infection with* Salmonella enterica* serovar Typhimurium [112]. Despite their preliminary nature, these findings indicate that lectins could well target bacterial organisms.

### 4.2. Yeasts and Other Fungi Are Potential Targets of Lectins

*Candida* normally lives harmless in several locations of the human body, including the skin and mucosal membranes, but can overgrow and cause a disease named candidiasis in the oral cavity, the gastrointestinal tract, and the genitalia [113]. For instance, in immunocompromised individuals the selective loss of Th17 cells with the progression of HIV infection causes the decay of fungal containment [114]. Interestingly,* Candida* spp. possess *β*-1,2 mannosylated glycoproteins (e.g.,* Candida* mannan) in addition to other sugars on their cell surface [115]. It is estimated that 80–90% of the cell wall protein mass in* Candida *are mannose residues added by N-glycosylation, O-glycosylation, and/or glycosylphosphatidylinositol (GPI) anchoring [116]. These sugar molecules are employed by the pathogen to initiate infection of the host cells in various mucosal surfaces. However,* Candida* spp. also adhere to inert abiotic surfaces, including intravascular and urinary catheters, prosthetic cardiac valves, and denture prostheses [117]. Interestingly,* Punica granatum* produces a chitin-binding lectin (PgTeL), with antifungal activity against* Candida albicans* and* C. krusei*, which are commonly found in immunocompromised individuals such as AIDS patients [118]. In addition, low dose of the plant lectin Con A has been shown to protect mice from a lethal dose of* C. albicans* by producing tumor necrosis factor *α* (TNF*α*) and activating macrophages, thus increasing the clearance of* C. albicans* [119]. SteLL, a chitin-binding lectin isolated from the leaves of the plant* Schinus terebinthifolius*, has shown antifungal activity against* C. albicans* at a minimal inhibitory concentration (MIC) of 6.5 *μ*g/ml [120]. Lectins isolated from legumes have also shown antifungal activity. For instance, ConBr from* Canavalia brasiliensis* and DvioL isolated from* Dioclea violacea* have both demonstrated antifungal activity against* C. albicans* at a MIC of 16 *μ*g/ml [121]. Another lectin, CasuL, alters the cell morphology and damages the cell wall in* Candida krusei*, indicating its potent antifungal properties [111].

Moreover, Lunatin, obtained from edible seeds of* Phaseolus lunatus* billb, shows potent antifungal activities against multiple fungal species, such as* Sclerotium rolfsii*,* Physalospora piricola*,* Fusarium oxysporum*, and* Botrytis cinerea *[122]. Taken together, the above findings demonstrate that lectins could interfere with the establishment of fungal diseases, though there is a need for further investigation.

### 4.3. Parasitic Organisms Potentially Affected by Lectins

The glycoconjugates of eukaryotic parasites, including N-glycans, O-glycans, and lipophosphoglycans, are critical to host-pathogen interactions, including adherence, invasion, and immune activation suggesting their potential importance in virulence [123].

Lipophosphoglycan associated attachment of trichomonads to the mucosal surface drive infection by* Trichomonas vaginalis*, commonly diagnosed in humans (170 million new infections/year) [123]. It is known that infection with* T. vaginalis* increases the risk of HIV acquisition [124]. Similar to HIV,* T. vaginalis* is sexually transmitted, meaning that using lectins with anti-HIV activity could potentially affect this parasite as well. Indeed, the well-known antiviral lectins CV-N and GRFT were recently evaluated for their effects on adherence of* Trichomonas vaginalis* to ectocervical cells as well as on* Tritrichomonas* infection in mice; treatment with the above lectins resulted in increased adherence of ricin-resistant mutants to ectocervical cells and organotypic EpiVaginal tissue cells, with decreased* Tritrichomonas *amounts recovered from the mouse vagina [125]. Although these effects were modest, they clearly provide a proof of concept that lectins could help tackle parasitic infections in humans.

*Toxoplasma gondii* is an obligate intracellular parasite that causes the disease known as toxoplasmosis. According to the CDC, more than 30 million individuals in the USA are* T. gondii* carriers [126]. Even though* Toxoplasma gondii* infection is asymptomatic in most people, pregnant women and immunocompromised individuals are at high risk of severe disease [127, 128]. Two lectins isolated from plants, including ScLL and ArtinM, have been shown to be beneficial in controlling* T. gondii* infection in mice [129]. The latter lectins induce the production of cytokines necessary to control* T. gondii* infection without any cytotoxicity. Additionally, they stimulate nitric oxide production by macrophages required for parasite killing. Eutirucallin, a plant lectin with broad antimicrobial properties, also showed antiparasitic activity* in vitro* against* T. gondii* [100].

## 5. Conclusion

Lectins are highly potent in neutralizing a very broad range of HIV strains and other enveloped viruses, with potential activity against multiple pathogenic prokaryotic and eukaryotic organisms that further complicate the HIV/AIDS problem. Therefore, these molecules deserve in-depth assessment for their inclusion in current efforts to end HIV infection. It should be noted that some lectins possess cytotoxicity and mitogenicity, which could potentially lead to severe adverse effects if used in humans. Meanwhile, most lectins are not monospecific and actually recognize a handful of different sugar types. Those recognizing glycans found on the surface of normal human cells have the potential to elicit off-target toxicity. However, comprehensive studies by our team and others have demonstrated the safety of GRFT. While still in preclinical development, lectins have shown tremendous potential to inhibit HIV and other pathogens and could be used to prevent multiple infections and/or improve the overall health status of HIV infected individuals.

## Figures and Tables

**Table 1:** Lectins with neutralization activities towards different enveloped viruses.

Virus	Antiviral lectins	EC50/IC50	References
Hepatitis C	Cyanovirin N (CV-N)	1.6–17.6 nM	[53]
	Griffithsin (GRFT)	13.9 nM	[54]
	Microcystis viridis (MVL)	30.4 nM	[55]
	Galanthus nivalis (GNA)	11.1 nM	[55]
	Cymbidium agglutinin (CA)	10 nM	[56]
	Hippeastrum hybrid agglutinin (HHA)	3 nM	[56]

Influenza A/B	Eucheuma serra (ESA-2)	12.4 nM	[57]
	Kappaphycus alvarezii (KAA-2)	12.3/1–10 nM	[58]
	Boodlea coasta (BCA)	18.8–74.2 nM	[59]
	Narcissus tazetta (NTL)	0.20 *μ*g/ml–1.33 *μ*g/ml	[60]

Herpes simplex types 1 and 2	Griffithsin (GRFT)	230 nM	[61]
	Cyanovirin N (CV-N)	Low nM	[62]
	Jackfruit lectin (JFL)	2.5 *μ*g/ml	[63]
	Typhonium divaricatum (L.) Decne	3.054 *μ*g/ml	[64]
	Polygonatum odoratum (POL)	2.5 *μ*g/ml	[65]

Japanese encephalitis virus	Griffithsin (GRFT)	20 nM	[50]

Coronavirus	Hippeastrum hybrid agglutinin (HHA)	3.2 *μ*g/ml	[66]
	Galanthus nivalis (GNA)	6.2 *μ*g/ml	[66]
	Cymbidium agglutinin (CA)	4.9 *μ*g/ml	[66]
	Urtica dioica agglutinin (UDA)	1.3 *μ*M	[67]

HIV	Griffithsin (GRFT)	0.04–0.63 nM	[45]
	BanLec	0.33–4.1 nM	[44]
	Actinohivin (AH)	2–110 nM	[42]
	Cyanovirin N (CV-N)	0.1–36.8	[19]
	Microvirin (MVN)	2.1–167 nM	[39]
